# Energy Harvesting Hybrid Acoustic-Optical Underwater Wireless Sensor Networks Localization

**DOI:** 10.3390/s18010051

**Published:** 2017-12-26

**Authors:** Nasir Saeed, Abdulkadir Celik, Tareq Y. Al-Naffouri, Mohamed-Slim Alouini

**Affiliations:** Department of Electrical Engineering, CEMSE Division, King Abdullah University of Science and Technology (KAUST), Thuwal 23955-6900, Makkah Province, Saudi Arabia; abdulkadir.celik@kaust.edu.sa (A.C.); tareq.alnaffouri@kaust.edu.sa (T.Y.A.-N.); slim.alouini@kaust.edu.sa (M.-S.A.)

**Keywords:** underwater sensor networks, acoustic-optical communication, energy harvesting, localization

## Abstract

Underwater wireless technologies demand to transmit at higher data rate for ocean exploration. Currently, large coverage is achieved by acoustic sensor networks with low data rate, high cost, high latency, high power consumption, and negative impact on marine mammals. Meanwhile, optical communication for underwater networks has the advantage of the higher data rate albeit for limited communication distances. Moreover, energy consumption is another major problem for underwater sensor networks, due to limited battery power and difficulty in replacing or recharging the battery of a sensor node. The ultimate solution to this problem is to add energy harvesting capability to the acoustic-optical sensor nodes. Localization of underwater sensor networks is of utmost importance because the data collected from underwater sensor nodes is useful only if the location of the nodes is known. Therefore, a novel localization technique for energy harvesting hybrid acoustic-optical underwater wireless sensor networks (AO-UWSNs) is proposed. AO-UWSN employs optical communication for higher data rate at a short transmission distance and employs acoustic communication for low data rate and long transmission distance. A hybrid received signal strength (RSS) based localization technique is proposed to localize the nodes in AO-UWSNs. The proposed technique combines the noisy RSS based measurements from acoustic communication and optical communication and estimates the final locations of acoustic-optical sensor nodes. A weighted multiple observations paradigm is proposed for hybrid estimated distances to suppress the noisy observations and give more importance to the accurate observations. Furthermore, the closed form solution for Cramer-Rao lower bound (CRLB) is derived for localization accuracy of the proposed technique.

## 1. Introduction

The development of underwater wireless communication capable sensor nodes has provided new opportunities for ocean exploration. The spatial-temporal data for oceanic exploration is of utmost importance for the applications of marine scientific research, ocean energy development and utilization of ecological underwater environment. Currently, the technologies used for underwater wireless communication consists of acoustic communication, electromagnetic waves communication, and optical communication. Acoustic communication systems have been one of the most used underwater wireless communication technology due to its ability to provide connectivity over very long distances. However, acoustic waves still have many drawbacks including scattering, high delay due to low propagation speeds, high attenuation, low bandwidth. Acoustic waves also have a bad impact on the health of underwater fishes and mammals. According to a recent report by natural resources defense council (NRDC), military sonar and other acoustic communication networks are rising the ocean noise, which has a serious impact on the health of underwater mammals [1,2].

Electromagnetic waves suffer from serious attenuation in water, the attenuation in the ocean is about 169 dB/m for the 2.4 GHz band, and the attenuation in freshwater is much higher, 189 dB/m [3]. Moreover, electromagnetic waves based underwater communication requires huge antennas and is limited to the shallow areas of the sea. On the other hand, operating at ultra-low frequencies yields reduced attenuation levels, in return for high hardware costs and low data rates. Underwater propagation of light waves also exhibits distinctive characteristics in different wavelengths as shown in [4]. In 1963, the authors [5] found that attenuation within the range of 450–550 nm wavelengths (blue and green lights) is much smaller compared to other wavelengths. In 1966, Gilbert et al. [6] experimentally confirmed this behavior of optical waves, which provided the foundation of underwater optical communication. Table 1 shows the comparison between three different kinds of underwater wireless communication carrier waves. Optical waves have the advantage of higher data rate and low power consumption but low transmission ranges. In this paper, a novel hybrid acoustic-optical underwater wireless sensor network (AO-UWSN) is considered that benefits from the advantages of both underwater acoustic communication and optical communication. While the optical wireless communication is employed to transmit at higher data for shorter range, the acoustic communication is used for the command and control within the long ranges, which does not require high data rates.

Notable research has been previously conducted on the energy consumption and lifetime maximization aspects of RF wireless sensor networks. In [7], the authors proposed an energy efficient routing protocol to transfer information in WSNs. Stable election protocol (SEP) and prolong stable election protocol (P-SEP) are proposed to balance the energy consumption in WSNs [8] and to increase the network stability [9]. Energy efficiency of acoustic UWSNs has been recently investigated in [10] for amplify-and-forward scheme along with a minimum energy routing protocol. An enhanced energy efficient protocol is addressed in [11], which also takes the depth of sensor nodes into account. A comprehensive survey is also presented on research challenges, localization schemes, and deployment of UWSNs in [12]. However, to the best of our knowledge, no literature exists that considers the energy efficiency, network lifetime, energy harvesting and localization of hybrid AO-UWSNs.

Today’s UWSNs consist of battery-powered sensor nodes with a limited energy budget. Albeit the notable research efforts on designing different protocols for underwater communication networks, no significant research has been carried out on the energy harvesting methods for UWOSNs. However, energy harvesting can play a significant role in performance enhancement and improve the lifetime of UWOSNs. Since underwater sensor nodes cannot survive on the battery power for a long time, energy harvesting is a promising solution to provide energy from the ambient sources in the aquatic environment. Noting that energy harvesting techniques used for terrestrial communications are not applicable for underwater sensor nodes in the aquatic environment, it is necessary to consider alternative energy harvesting methods, e.g., acoustic piezo-electric harvesters [13] and microbial fuel cells [14].

The underwater aquatic monitoring demands accurate localization techniques as the collected data is only useful if the location of the nodes is known to a certain accuracy. Nonetheless, localization of sensors within UWSNs is also a crucial and challenging task especially for the surveillance applications, which can scale for a large number of sensor nodes. A number of acoustic underwater sensor networks’ localization techniques based on time difference of arrival (TDoA) has already been proposed, which consider different parameters such as signal propagation model, network topology, environmental factors, localization accuracy, number of anchor nodes, the geometry of anchor nodes and the relative location of the sensor node to the anchors [15,16,17,18,19,20,21]. However, the TDoA measurements for distance estimation in underwater acoustic communication channels are highly affected by multipath. Received signal strength (RSS) based distance estimation did not get much attention for UWSNs’ localization since the multipath propagation of acoustic communication makes it hard to achieve accurate distance estimation [22]. However, the underwater acoustic channels show good transmission features at certain water depths and RSS based distance estimation can be considered [23]. In this paper, we propose RSS based AO-UWSNs’ localization with the capability of energy harvesting.

The contributions of this paper are summarized as follows:

1. A novel hybrid acoustic-optical underwater wireless sensor network localization technique is proposed in order to benefit from the advantages of both acoustic and optical communication.
2. A weighted multiple observations paradigm is proposed for hybrid estimated distances to suppress the noisy observations and give more importance to accurate observations. Considering the hybrid acoustic and optical RSS model, the closed form solution for Cramer–Rao lower bound (CRLB) is also derived to improve the localization accuracy of the proposed technique.
3. We consider energy harvesting for the battery limited sensor nodes and show the impact of harvested energy on the network lifetime maximization and energy efficiency.

### 1.1. Notations and Symbols

We have used the following notations: Matrices and vectors are denoted by boldface upper-case and boldface lower-case letters and symbols, respectively. Scalars are denoted by non-boldface italic letters and symbols. Superscripts ${\left(\cdot \right)}^{T}$, ${\left(\cdot \right)}^{+}$ and ${\left(\cdot \right)}^{-1}$ denote the transpose, Moore-Penrose pseudoinverse and inverse, respectively. For their convenience, we refer readers to Table 2 for the list of symbols used throughout the paper.

### 1.2. Paper Organization

The remainder of the paper is organized as follows: The system model and problem formulation for hybrid AO-UWSNs are presented in Section 2. In Section 2.4, the proposed localization technique is devised for AO-UWSNs. In Section 3, we analyze the performance of the proposed technique by deriving its CRLB. Section 4 and Section 5 present the numerical results and conclusions, respectively.

## 2. System Model and Proposed Technique

In this section, the system model and proposed localization technique are introduced.

### 2.1. System Model

Consider an AO-UWSN which consists of *m* anchor nodes and *n* sensor nodes embedded on the ocean floor or suspending sensors. Assuming that m<n and anchor node locations are known apriori, a fully connected network is considered where each sensor node is able to communicate with close and distant neighbors using optical and acoustic channels, respectively. Every sensor node shares its neighborhood information with the surface buoy. The proposed technique consists of three major steps:

- **Step 1:** The sensor nodes sweep the neighboring region using the optical channel and find the ranges to its neighbors.
- **Step 2:** Nodes which are not within the reach of an optical channel are communicated by using the acoustic channel and computes the acoustic ranges.
- **Step 3:** The surface buoy fuses the optical and acoustic ranges to compute the pairwise estimated distance matrix and applies a weighted multiple observation dimensionality reduction to find out the location of each sensor node.

### 2.2. Acoustic Underwater Ranging

Underwater acoustic channels suffer from two kinds of major losses: attenuation loss and spreading loss [24]. Attenuation loss is a result of scattering, diffraction, absorption, and leakage from ducts while spreading loss is a combination of cylindrical and spherical losses [25]. In general, the sea water transmission loss between any two nodes *i* and *j* is modeled as

(1) $$\eta_{t}=\eta_{s}+\eta_{c}+10^{-3}\alpha d_{ij},$$

where $\eta_{s}$ is spherical spreading loss, $\eta_{c}$ is the cylindrical spreading loss, α is the absorption coefficient, and $d_{ij}$ is the Euclidean distance between nodes *i* and *j*. Using the Thorp absorption model [26], α only depends on the frequency *f*, i.e.,

(2) $$\alpha =\frac{0.11f^{2}}{1+f^{2}}+\frac{44f^{2}}{4100+f^{2}}.$$

Spherical spreading loss fits the measured data appropriately. Therefore, ignoring the cylindrical loss and substituting α in (1) for spherical losses $\eta_{s}=20\log\left(d_{ij}\right)$, we get

(3) $$\eta_{t}=20\log\left(d_{ij}\right)+10^{-3}\left(\frac{0.11f^{2}}{1+f^{2}}+\frac{44f^{2}}{4100+f^{2}}\right)d_{ij}.$$

**Figure 1:**
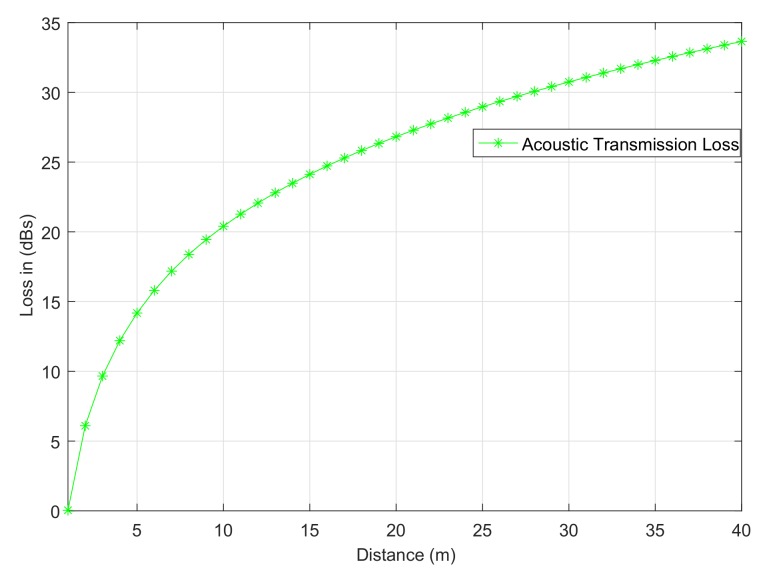
Acoustic transmission loss vs. distance.

Figure 1 shows the transmission loss between any two nodes *i* and *j* with respect to distance. The acoustic ranging based distance ${\tilde{d}}_{a_{ij}}$ is estimated from (3) using the real part of Lambert $W_{0}$ function [27]

(4) $${\tilde{d}}_{a_{ij}}=\frac{20000W_{0}\left(1.15\exp^{-4}\alpha \exp^{0.11\eta_{t}}\right)}{2.3\alpha }.$$

### 2.3. Optical Underwater Ranging

Optical light passing through the aquatic medium suffers from widening and attenuation in angular, temporal, and spatial domains [28]. The widening and attenuation of the underwater optical signals are dependent on the wavelength. Combining the absorption coefficient a(λ) and scattering coefficient s(λ) results in the extinction coefficient [29] defined as

(5) e(λ)=a(λ)+s(λ).

The propagation loss is the function of distance $d_{ij}$ and extinction coefficient e(λ) and is given by

(6) $${Ł}_{ij}=\exp^{-e\left(\lambda \right)d_{ij}}.$$

In this paper, we consider line of sight communication, where the sensor node *i* directs the optical light to sensor node *j*. Then, the received power at sensor node *j* is given as [30],

(7) $$P_{r_{j}}=P_{t_{i}}\eta_{i}\eta_{j}{Ł}_{ij}\frac{A_{j}\cos\theta }{2\pi d_{ij}^{2}\left(1-\cos\theta_{0}\right)},$$

where $P_{t_{i}}$ is the optical power transmitted by node *i*, $\eta_{i}$ and $\eta_{j}$ are the optical efficiencies of node *i* and *j*, respectively, $A_{j}$ is the aperture area of node *j*, θ is the angle between trajectory of node *i* transmitter and node *j*, and $\theta_{0}$ is the divergence angle of the transmitted signal. The most common modulation method for optical wireless communications in the literature is the intensity modulation with direct detection (IM/DD). The bit error rate (BER) expression for IM/DD with on-off keying is developed for the number of a photon arriving at the photon counter using Poisson model. The number of photons arriving at node *j* in time duration *T* is

(8) $$\varrho =\frac{P_{r_{j}}\eta_{j}\lambda }{TR_{d}h\overset{´}{c}},$$

where $R_{d}$ is the data rate, *h* is the Planck’s constant and $\overset{´}{c}$ is the free space speed of light. The BER for the photons arriving at node *j* is expressed as

(9) $$b_{j}=\frac{1}{2}\mathrm{erfc}\left(\sqrt{\frac{T}{2}\left(\sqrt{r_{1}}-\sqrt{r_{0}}\right)}\right),$$

where $r_{1}=r_{d}+r_{j}+r_{b}$ and $r_{0}=r_{d}+r_{b}$ represent the photons required for transmission of binary 1 and 0 respectively, while $r_{d}$ is the dark count noise and $r_{b}$ is the noise produced due to background illumination. Substituting the values of $r_{1}$ and $r_{0}$ in (9) and solving for $r_{j}$ yields

(10) $$r_{j}={\left(\sqrt{r_{d}+r_{b}}+\sqrt{\frac{2}{T}}{\mathrm{erfc}}^{-1}\left(2b_{j}\right)\right)}^{2}-r_{d}-r_{b}.$$

Substituting (7) and (8) in (10), then estimated optical distance ${\tilde{d}}_{o_{ij}}$ between node *i* and *j* is obtained as [30],

(11) $${\tilde{d}}_{o_{ij}}=\frac{2\cos\theta }{e(\lambda )}W_{0}\left(\frac{e(\lambda )}{2\cos\theta \sqrt{\frac{2\pi Th\overset{´}{c}R_{d}r_{j}\left(1-\cos\theta_{0}\right)}{\eta_{j}\lambda P_{t_{i}}\eta_{i}\eta_{j}A_{j}\cos\theta }}}\right),$$

where $W_{0}\left(.\right)$ is the real part of Lambert *W* function.

### 2.4. Proposed Localization Technique

Given that the noisy range measurements $\Lambda ={\left\{{\tilde{d}}_{ij}\right\}}_{i,j=1,i\neq j}^{m+n}$ are available from (4) and (11), we define our problem as finding the configuration of m+n nodes in lower dimensional space from ${\tilde{d}}_{ij}$ such that it is well approximated by the $d_{ij}$. The error function for the pairwise noisy distances is defined as

(12) $$\min_{L}\sum_{i<j}v_{ij}{\left({\tilde{d}}_{ij}-d_{ij}\left(L\right)\right)}^{2},$$

where $\left(L\right)=\left\{l_{1},l_{2},\ldots ,l_{m+n}\right\}$ are the two-dimensional coordinates of all the nodes and $v_{ij}$ are the weighting coefficients. A number of different techniques are available in the literature to solve this optimization problem, but all of them consider a single observations approach. In this paper, we propose a dimensionality reduction technique with multiple input observations. The objective function for the proposed technique is defined

(13) $$\min_{\Phi^{\left(u\right)},L}\sum_{u=1}^{R}\Phi^{{\left(u\right)}^{\gamma }}\sum_{i<j}v_{ij}{\left({\tilde{d}}_{ij}-d_{ij}\left(L\right)\right)}^{2},$$

where $\Phi^{\left(u\right)}$ is the importance of *u*-th observation such that $\sum_{u=1}^{R}\Phi^{\left(u\right)}=1$, γ is the controlling parameter and *R* is the total number of observations. The controlling parameter for weights (γ>1) determine the distribution of the multiple observations. The weight of each observation is added to the error function in (13). The logic behind using multiple observations is that, if $\Phi^{\left(u\right)}$ is used directly, then the observation view with small error function has $\Phi^{\left(u\right)}=1$, and the rest of the observation views have $\Phi^{\left(u\right)}=0$. This behavior is not optimal as only one view is selected while all other views are ignored. Therefore, the proposed adaptive weight learning paradigm is an intuitive solution that considers a combination of weights for each observation view. The optimization problem in (13) is solved by decomposing it into two sub-problems.

#### 2.4.1. Updating L for fixed $\Phi^{\left(u\right)}$

In the first sub-problem, (13) is rewritten as

(14) $$\min_{L}\left(\rho_{1}+\rho_{1}-2\rho_{3}\right),$$

where

(15) $$\rho_{1}=\sum_{u=1}^{R}\sum_{i<j}\Phi^{{\left(u\right)}^{\gamma }}v_{ij}{\tilde{d}}_{ij}^{{\left(u\right)}^{2}},$$

(16) $$\rho_{2}=\sum_{u=1}^{R}\sum_{i<j}\Phi^{{\left(u\right)}^{\gamma }}v_{ij}d_{ij}^{2}{\left(L\right)}^{\left(u\right)},$$

and

(17) $$\rho_{3}=\sum_{u=1}^{R}\sum_{i<j}\Phi^{{\left(u\right)}^{\gamma }}v_{ij}{\tilde{d}}_{ij}^{\left(u\right)}d_{ij}{\left(L\right)}^{\left(u\right)}.$$

Optimization of this sub-problem is achieved by a majorization approach. It is clear from (14) that the first term $\rho_{1}$ is dependent on fixed weights $v_{ij}$ and constant estimated distance ${\tilde{d}}_{ij}$, thus ignored from the optimization process. The second term in (14) is the sum of weighted squared distances, which is written as

(18) $$\rho_{2}=Tr\left(L^{T}\left(v_{ij}\Phi^{{\left(u\right)}^{\gamma }}\right)L\right)=Tr\left(L^{T}\Theta L\right),$$

where $\Theta \in R^{\left((m+n)\times (m+n)\right)}$ with elements

(19) $$\Theta_{ij}=\left\{\begin{array}{cc} -\sum_{u=1}^{R}\Phi^{{\left(u\right)}^{\gamma }}v_{ij}, & \mathrm{if}\;i\neq j,\\ \sum_{j=1,j\neq i}^{m+n}\sum_{u=1}^{R}\Phi^{{\left(u\right)}^{\gamma }}v_{ij}, & \mathrm{if}\;i=j. \end{array}\right.$$

The last term in (14) is the sum of weighted distances for all the observations, by Cauchy–Shwartz inequality $\rho_{3}$ is approximated as

(20) $$\rho_{3}=\sum_{i<j}\left(\sum_{u=1}^{R}\Phi^{{\left(u\right)}^{\gamma }}v_{ij}{\tilde{d}}_{ij}^{\left(u\right)}\right)d_{ij}\left(L\right)\leq Tr\left(L^{T}BZ\right),$$

where Z are the estimated points from previous iteration and the elements of B are defined as

(21) $$b_{ij}=\left\{\begin{array}{cc} -\frac{\sum_{u=1}^{R}\Phi^{{\left(u\right)}^{\gamma }}v_{ij}{\tilde{d}}_{ij}^{\left(u\right)}}{d_{ij}(Z}, & \mathrm{if}\;i\neq j\;\mathrm{and}\;d_{ij}(Z\neq 0),\\ 0, & \mathrm{if}\;i\neq j\;\mathrm{and}\;d_{ij}(Z=0), \end{array}\right.$$

and

(22) $$b_{ii}=\sum_{j=1,j\neq i}^{m+n}b_{ij}.$$

Substituting the values of $\rho_{1}$, $\rho_{2}$ and $\rho_{3}$ in the objective function defined in (14) yields

(23) $$\min_{L}\left(\rho_{1}+\rho_{1}-2\rho_{3}\right)=\rho_{1}+Tr\left(L^{T}\Theta L\right)-2\cdot Tr\left(L^{T}BZ\right).$$

The minimum of function in (23) is calculated by taking its partial derivative with respect to L as

(24) $$\frac{\partial \chi }{\partial L}=2\Theta L-2BZ.$$

By setting (24) to zero, we get

(25) $$\hat{L}=\Theta^{+}BZ,$$

where $\Theta^{+}$ represents the Moore–Penrose inverse of Θ. There are no missing elements in matrix Θ therefore, (25) is simplified as

(26) $$\hat{L}=\frac{1}{\left(m+n\right)\sum_{u=1}^{R}\Phi^{{\left(u\right)}^{\gamma }}}BZ.$$

The estimated locations in (26) can be refined using any linear transformation method with the help of anchors. Here we used Helmert transformation [31] for transformation from local coordinates to global coordinates, i.e.,

(27) $$\tilde{L}=\kappa \Omega^{T}\left(\hat{L}\right)+\Psi ,$$

where κ, Ω and Ψ are the scaling, rotation, and translation factors for coordinates transformation, respectively.

#### 2.4.2. Updating $\Phi^{\left(u\right)}$ for Fixed L

In order to simplify the notation, (13) is re-written as

(28) $$\rho =\sum_{u=1}^{R}\Phi^{{\left(u\right)}^{\gamma }}\rho^{u},$$

where $\rho^{u}=\sum_{i<j}v_{ij}{\left({\tilde{d}}_{ij}-d_{ij}\left(L\right)\right)}^{2}$ is the *u*-th observation. To solve this sub-problem, we use Lagrange multiplier by taking the assumption that $\sum_{u=1}^{R}\Phi^{\left(u\right)}=1$. The Lagrangian function of ρ is

(29) $$L\left(\rho ,\bar{\lambda }\right)=\sum_{u=1}^{R}\Phi^{{\left(u\right)}^{\gamma }}\rho^{u}+\bar{\lambda }\left(\sum_{u=1}^{R}\Phi^{\left(u\right)}-1\right),$$

where $\bar{\lambda }$ is the rate of change of the function. The partial derivative of (29) with respect to $\Phi^{\left(u\right)}$ is

(30) $$\frac{\partial L(\rho ,\bar{\lambda })}{\partial \Phi^{\left(u\right)}}=\gamma \Phi^{{\left(u\right)}^{\left(\gamma -1\right)}}\rho^{u}-\bar{\lambda }.$$

By setting (30) equal to zero, we get

(31) $$\Phi^{\left(u\right)}={\left(\frac{\bar{\lambda }}{\gamma \rho^{u}}\right)}^{\frac{1}{\bar{\lambda }-1}}.$$

Using the constraint of $\sum_{u=1}^{R}\Phi^{\left(u\right)}=1$, the multiplier term $\bar{\lambda }$ is dropped out and the optimal value of $\Phi^{\left(u\right)}$ is obtained as

(32) $$\Phi^{\left(u\right)}=\frac{\rho^{{\left(u\right)}^{\frac{1}{1-\gamma }}}}{\sum_{u=1}^{R}\rho^{{\left(u\right)}^{\frac{1}{1-\gamma }}}}.$$

Note that the choice of γ in (32) depends on the correlation between different observation views. If the observation views are highly correlated to each other, then a large value of γ is preferred because this results in getting equal weights. But if the observation views are highly uncorrelated then small value of γ is selected because this results in giving more importance to the accurate observation views.

#### 2.4.3. Impact of Energy Harvesting on Localization Performance

The energy consumed by all the nodes in the network is expressed as

(33) $$E_{t}=\sum_{i=1}^{m+n}E_{f_{i}}+\left(m+n\right)\sum_{i=1}^{m+n}E_{r_{i}},$$

where $E_{f_{i}}$ is the energy required for basic operation of the electronic circuitry and $E_{r_{i}}$ is the energy consumed for transmission. $E_{r_{i}}$ can be expressed as

(34) $$E_{r_{i}}=E_{b}{\left(\frac{4\pi r_{i}}{\lambda^{\prime }}\right)}^{2},$$

where $E_{b}$ is the energy required for single bit transmission, $r_{i}$ is the transmission range of node *i* and $\lambda^{\prime }$ is the wavelength. The energy consumption is directly proportional to the square of the transmission range $r_{i}$. Since the accuracy of the localization technique and the transmission range $r_{i}$ depend on the energy arrival into the network, the efficiency η of the localization technique can be defined as

(35) $$\eta =\frac{E_{t}}{\delta^{2}},$$

where $\delta^{2}=E\left(\left(\tilde{l}-l\right){\left(\tilde{l}-l\right)}^{T}\right)$ is the mean square error of a single node localization. As a result, the efficiency of the proposed localization technique increases with the energy arriving from the ambient underwater energy sources.

#### 2.4.4. Complexity Analysis of the Proposed Technique

In order to find the location of given sensor nodes, the first step is to find all the pairwise distances. Then, a procedure should be followed to minimize the discrepancy between the pairwise distances and their actual Euclidean distances in a low-dimensional space. To find the pairwise distances between non-neighboring nodes, single hop local distances or numerical methods for distance estimation are used [32,33]. The time complexity to find out all the pairwise distances is $O(Z^{3})$, where Z=m+n are the total number of nodes. The time complexity of the proposed multiple observation technique to compute the pairwise distances is on the order of $O(Z^{3})$. The transformation from the local coordinates to global coordinates using anchor nodes requires $O\left(m^{2}\right)+O\left(Z\right)$ amount of time. Thus, the total complexity of the proposed technique is

(36) $$\mathrm{Complexity}=O\left(Z^{3}\right)+O\left(m^{2}\right)+O\left(Z\right),$$

which can be regarded as $O(Z^{3})$ as the overall complexity is dominated by the first term.

## 3. Performance Analysis

The Cramer–Rao lower bound (CRLB) defines the lower bound for the variance of any unbiased estimator, when the range measurement error is zero mean Gaussian distributed [34]. The probability density function for ${\tilde{d}}_{ij}$, conditioned on $l_{i}$ and $l_{j}$, can be written as

(37) $$\begin{array}{c} f\left({\tilde{d}}_{ij}|l_{i},l_{j}\right)=\frac{1}{\sqrt{2\pi \sigma_{ij}^{2}}}e^{\left(-\frac{1}{2\sigma_{ij}^{2}}{\left({\tilde{d}}_{ij}-d_{ij}\right)}^{2}\right)}, \end{array}$$

where ${\tilde{d}}_{ij}$ is the estimated distance, $d_{ij}=∥l_{i}-l_{j}∥=\sqrt{{\left(x_{i}-x_{j}\right)}^{2}+{\left(y_{i}-y_{j}\right)}^{2}}$ is the Euclidean distance, and $\sigma_{ij}^{2}$ is the noise variance. The expression for the Fisher information matrix (FIM) becomes

(38) $$I\left(l\right)=E_{f(\tilde{d}/l)}\left\{{\left[\frac{\partial \ln(f\left(\tilde{d}/l\right))}{\partial l}\right]}^{T}\left[\frac{\partial \ln(f\left(\tilde{d}/l\right))}{\partial l}\right]\right\}.$$

It is assumed that the noise added to the ranging measurements is zero mean Gaussian process with variance $\sigma_{\tilde{d}}$. Therefore, the RSS based noise co-variance matrix is $\Gamma_{\tilde{d}}=\mathrm{diag}\left(\sigma_{{\tilde{d}}_{1}},\sigma_{{\tilde{d}}_{2}},\ldots ,\sigma_{{\tilde{d}}_{n}}\right)$. Then, the likelihood ratio is computed as

(39) $$\begin{array}{ccc} \ln(f\left(\tilde{d}/l\right)) & = & \ln\left(\frac{1}{{\left(2\pi \Gamma_{\tilde{d}}\right)}^{\frac{\left(n\right)}{2}}}\right)\\ & & -\frac{1}{2}{\left(\tilde{d}-d\left(l\right)\right)}^{T}\Gamma_{\tilde{d}}^{-1}\left(\tilde{d}-d\left(l\right)\right). \end{array}$$

The FIM is constructed from the likelihood ratios, given by

(40) $$\chi_{\tilde{d}}=\Theta_{d}^{T}\Gamma_{d}^{-1}\Theta_{d},$$

where

(41) $$\Theta_{d}=-\beta {\left[\begin{array}{c} \frac{x_{i}-x_{j}}{d^{2}}\\ \frac{y_{i}-y_{j}}{d^{2}} \end{array}\right]}^{T},$$

and β is the path loss exponent. The elements of $\chi_{\tilde{d}}$ are derived as

(42) $${\left\{\chi_{\tilde{d}}\right\}}_{1,1}=\frac{▽{\left(\tilde{d}-d\left(l\right)\right)}^{T}}{▽x}\Gamma_{d}^{-1}\frac{▽\left(\tilde{d}-d\left(l\right)\right)}{▽x},$$

(43) $${\left\{\chi_{\tilde{d}}\right\}}_{1,2}=\frac{▽{\left(\tilde{d}-d\left(l\right)\right)}^{T}}{▽x▽y}\Gamma_{d}^{-1}\frac{▽\left(\tilde{d}-d\left(l\right)\right)}{▽x▽y},$$

and

(44) $${\left\{\chi_{\tilde{d}}\right\}}_{2,2}=\frac{▽{\left(\tilde{d}-d\left(l\right)\right)}^{T}}{▽y}\Gamma_{d}^{-1}\frac{▽\left(\tilde{d}-d\left(l\right)\right)}{▽y}.$$

Simplifying (42)–(44), we get

(45) $${\left\{\chi_{\tilde{d}}\right\}}_{1,1}=\frac{\beta^{2}{\left(x_{i}-x_{j}\right)}^{T}\Gamma_{d}^{-1}\left(x_{i}-x_{j}\right)}{d^{4}},$$

(46) $${\left\{\chi_{\tilde{d}}\right\}}_{1,2}=\frac{\beta^{2}{\left(x_{i}-x_{j}\right)}^{T}\Gamma_{d}^{-1}\left(y_{i}-y_{j}\right)}{d^{4}},$$

and

(47) $${\left\{\chi_{\tilde{d}}\right\}}_{2,2}=\frac{\beta^{2}{\left(y_{i}-y_{j}\right)}^{T}\Gamma_{d}^{-1}\left(y_{i}-y_{j}\right)}{d^{4}}.$$

The CRLB is computed as the inverse of the diagonal elements of the FIM, i.e.,

(48) $$\mathrm{CRLB}={\left\{\chi_{\tilde{d}}\right\}}_{1,1}^{-1}+{\left\{\chi_{\tilde{d}}\right\}}_{2,2}^{-1}.$$

Finally, the mean square error of each node should satisfy the following condition:

(49) $$\frac{\sum_{i=1}^{n}{\left(l_{i}-{\tilde{l}}_{i}\right)}^{2}}{n}\geq {\left\{\chi_{\tilde{d}}\right\}}_{1,1}^{-1}+{\left\{\chi_{\tilde{d}}\right\}}_{2,2}^{-1}.$$

The condition in (49), gives the theoretical lower accuracy bound for the proposed localization technique which is valuable to design a localization system.

## 4. Numerical Results

**Figure 2:**
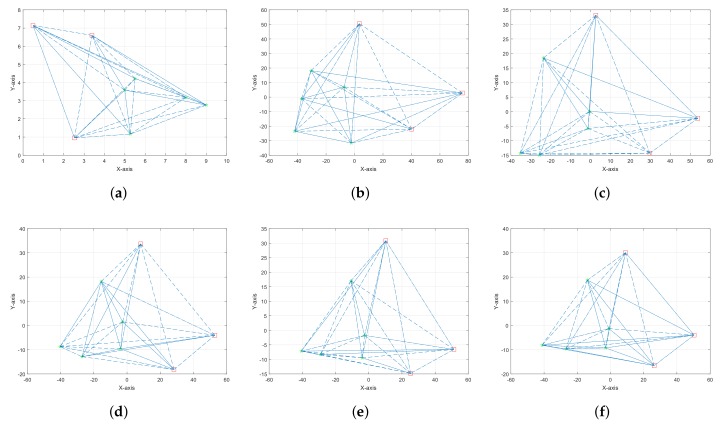
(**a**) Actual locations of the nodes for *m* = 3 and *n* = 5; (**b**) Single observation at $\sigma^{2}$ = 1.77 m; (**c**) Single observation at $\sigma^{2}$ = 0.5 m; (**d**) Single observation at $\sigma^{2}$ = 0.17 m; (**e**) Single observation at $\sigma^{2}$ = 0.05 m; (**f**) Multiple observations.

In this section, we present numerous simulation results executed in MATLAB (R2016b, MathWorks, Inc., Natick, MA, USA) to investigate the performance of proposed AO-UWSNs localization technique. Two different scenarios are generated in a square area of $\left(10\times 10\right)\;m^{2}$ for a various number of anchor nodes and sensor nodes. To generate different observations for each scenario, we randomly select m+n pair of noisy ranges $\tilde{d_{ij}}$. Gaussian noise is added to each $\tilde{d_{ij}}$, with mean $d_{ij}$ and variance $\sigma^{2}$. The first scenario consists of three anchor nodes (red squares) and five sensor nodes (green stars) as shown in Figure 2a. Four different observation views are generated for each scenario as shown in Figure 2b–e. It is clear from Figure 2f that the multiple observations result in smaller error function. The setup parameters for the first scenario and comparison of the error function are shown in Table 3.

**Figure 3:**
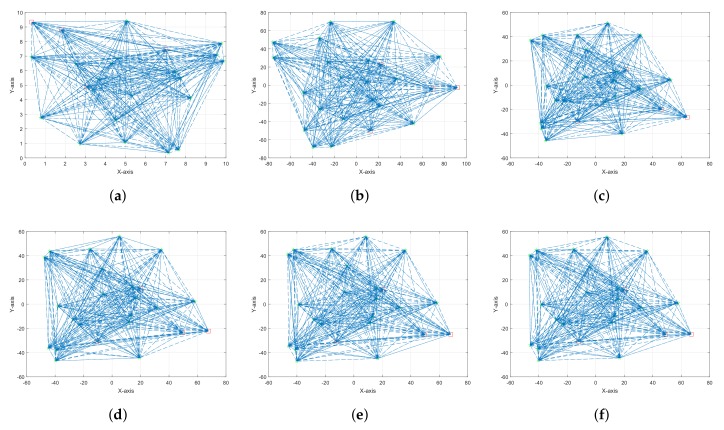
(**a**) Actual locations of the nodes for *m* = 4 and *n* = 20; (**b**) Single observation at $\sigma^{2}$ = 1.56 m; (**c**) Single observation at $\sigma^{2}$ = 0.5 m; (**d**) Single observation at $\sigma^{2}$ = 0.15 m; (**e**) Single observation at $\sigma^{2}$ = 0.04 m; (**f**) Multiple observations.

**Figure 4:**
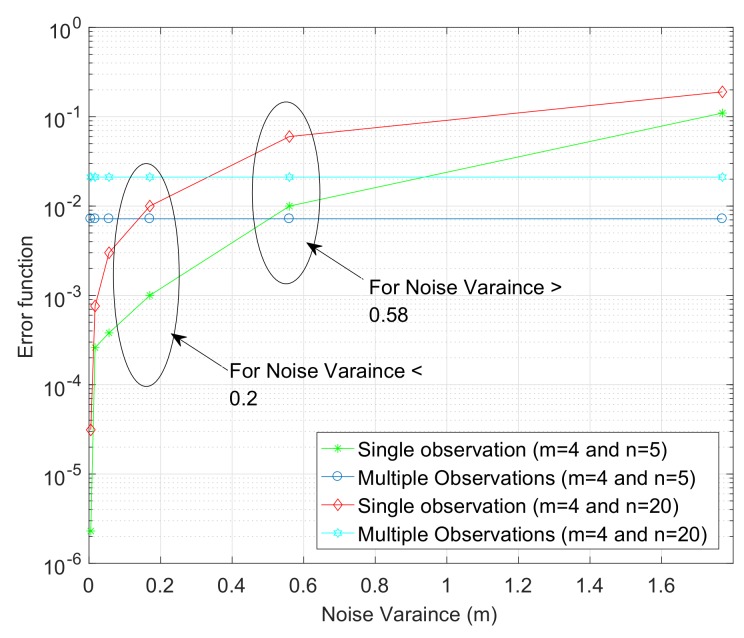
Error function vs. noise variance for single and multiple observations.

**Figure 5:**
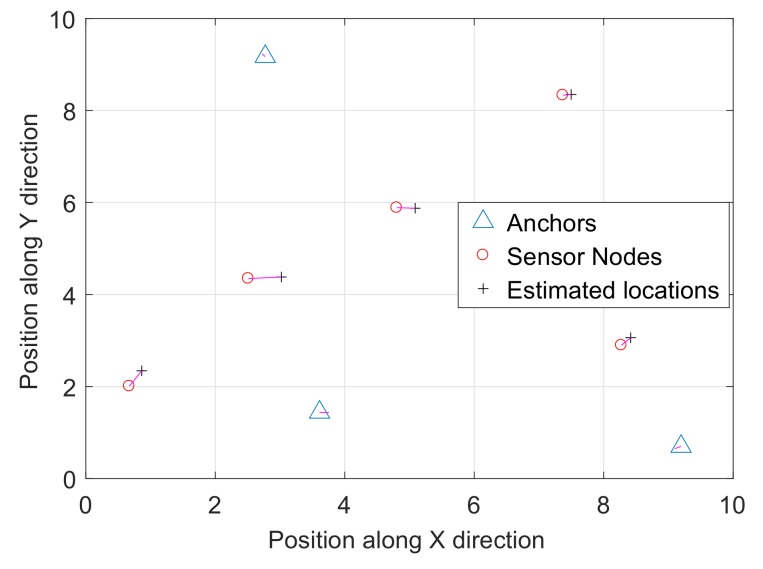
Mean square error for *m* = 3 and *n* = 5.

**Figure 6:**
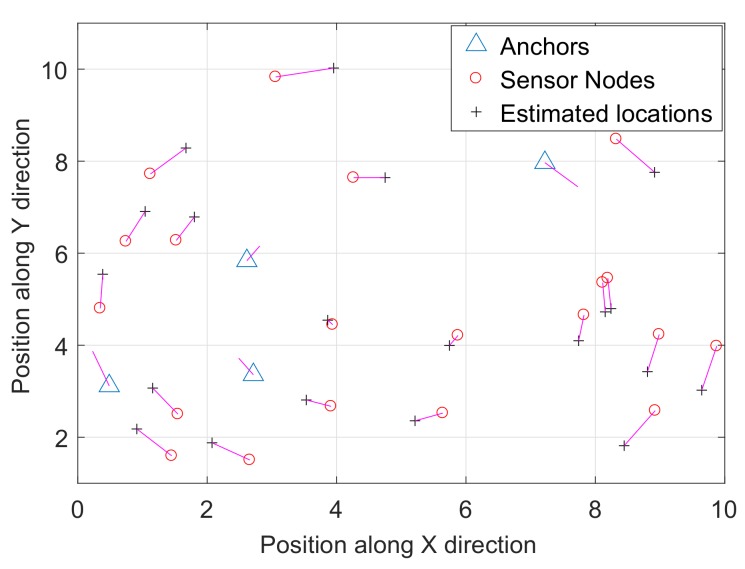
Mean square error for *m* = 4 and *n* = 20.

The second scenario consists of four anchor nodes and 20 sensor nodes randomly deployed in a square area of $\left(10\times 10\right)\;m^{2}$ as shown in Figure 3a. Figure 3b–e shows the results of four different single observation views with different noise variance. It is clear from Figure 3f that the multiple observations result in a smaller error function. The setup parameters for the second scenario and comparison of the error function are shown in Table 4. The comparison for the two different scenarios between a single observation and multiple observations is shown in Figure 4, which clearly tells us that the proposed multiple observations approach results in a better approximation of the sensor nodes’ configuration for a large noise variance. The final location estimation for both of the scenarios are achieved by using the anchor nodes, and Figure 5 and Figure 6 show the final location estimation of the first and second scenarios with a mean square error of 0.12 m and 0.64 m, respectively.

**Figure 7:**
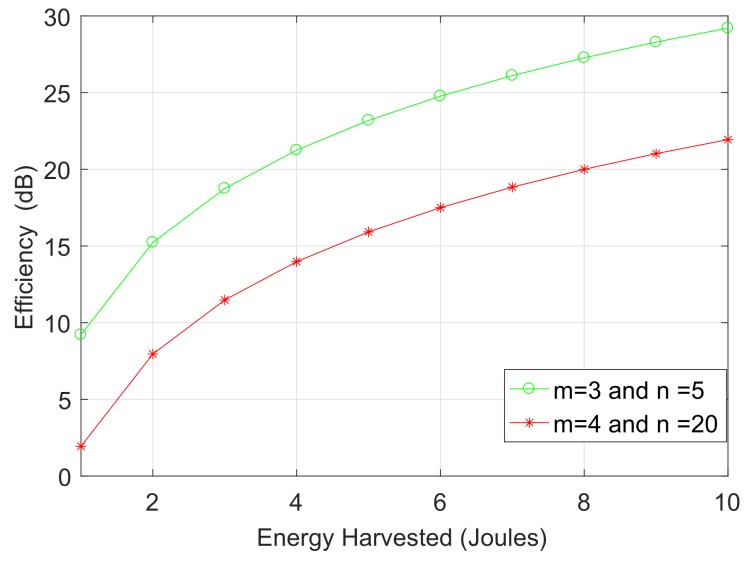
Efficiency vs. energy harvested.

**Figure 8:**
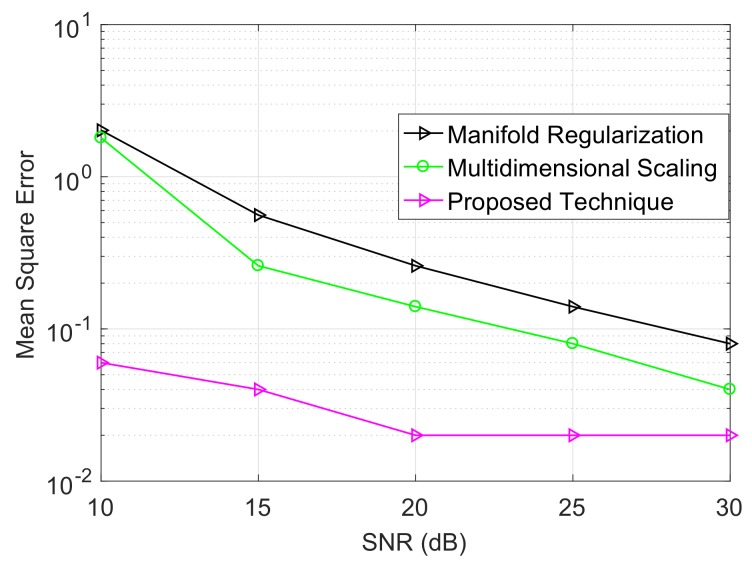
Signal to noise ratio (SNR) vs. mean square error.

The energy efficiency of a localization technique is characterized by (35), and Figure 7 shows the impact of energy harvested with respect to the efficiency for a target mean square error. The target means that the square error is set to 0.12 m and 0.64 m for first and second scenario, respectively. It is clear from Figure 7 that the efficiency of the localization technique improves with the energy harvested from the aquatic environment. The mean square error performance of the proposed technique is compared with well-known network localization schemes such as multidimensional scaling [35] and manifold regularization [36]. Figure 8 shows that the proposed technique outperforms both multidimensional scaling and manifold regularization due to the novel strategy of weighting the multiple observation views for a single network.

## 5. Conclusions

In this paper, a novel hybrid acoustic-optical underwater wireless sensor network localization technique is proposed, in order to get the advantage of both acoustic and optical communication. An energy harvesting scheme is devised for the battery limited sensor nodes to increase the lifetime of the network. A weighted multiple observations paradigm is proposed for hybrid estimated distances to suppress the noisy observations and give more importance to accurate observations. A number of simulations are conducted to verify the performance of the proposed localization technique.

## Figures and Tables

**Table 1:** Underwater wireless communication channels comparison.

Parameters	EM Waves	Acoustic Waves	Optical Waves
Communication Distance	100 m	Upto 20 Km	10–100 m
Transmit Power	Few mW to Hundred of Watts	10–100 W	Few Watts
Cost	High	High	Low
Data Rate	Up to 100 Mbps	In Kbps	Up to Gbps

**Table 2:** List of symbols.

Symbol	Variable	Symbol	Variable
*m*	Number of anchor nodes	$\sigma_{ij}$	Noise variance
*n*	Number of sensor nodes	$\theta_{0}$	Divergence angle
$\eta_{s}$	Spherical spreading loss	*T*	Time duration
$\eta_{c}$	Cylindrical spreading loss	l	Actual two-dimensional location of a node
α	Absorption coefficient	Λ	Matrix of Estimated distances
$d_{ij}$	Euclidean distance	$v_{ij}$	Weighting coefficients
${\tilde{d}}_{ij}$	Estimated distance	Φ	Importance of an observation
λ	Wavelength	γ	Controlling parameter
e(λ)	Extinction coefficient	L	Actual locations of all the nodes
$r_{j}$	Number of photons	$\tilde{L}$	Estimated locations of all the nodes
${Ł}_{ij}$	Propagation loss	κ	Scaling factor
$P_{r_{j}}$	Received power at node *j*	Ω	Rotation factor
$P_{t_{i}}$	Transmitted power by node *i*	Ψ	Translation factor
$\eta_{i},\;\eta_{j}$	Optical efficiencies	$E_{t}$	Energy consumption
θ	Trajectory angle	$\Gamma_{\tilde{d}}$	Noise co-variance matrix
*r*	Transmission range	$\delta^{2}$	Mean square error
${\tilde{d}}_{a_{ij}}$	Estimated acoustic distance	${\tilde{d}}_{o_{ij}}$	Estimated optical distance

**Table 3:** Parameters setup and error function comparison for the first scenario.

Observations	*m*	*n*	$\sigma^{2}$	Error Function
1st	3	5	1.77 m	0.11
2nd	3	5	0.56 m	0.01
3rd	3	5	0.17 m	0.001
4th	3	5	0.05 m	2.3 $\times 10^{-4}$
Multiple	3	5	-	2.3 $\times 10^{-6}$

**Table 4:** Parameters setup and error function comparison for second scenario.

Observations	*m*	*n*	$\sigma^{2}$	Error Function
1st	4	20	1.56 m	0.19
2nd	4	20	0.49 m	0.06
3rd	4	20	0.15 m	0.018
4th	4	20	0.04 m	0.0036
Multiple	4	20	-	9.6 $\times \;10^{-6}$
